# Branched-chain fatty acids affect the expression of fatty acid synthase and C-reactive protein genes in the hepatocyte cell line

**DOI:** 10.1042/BSR20230114

**Published:** 2023-10-31

**Authors:** Paulina Gozdzik, Aleksandra Czumaj, Tomasz Sledzinski, Adriana Mika

**Affiliations:** 1 Department of Pharmaceutical Biochemistry, Faculty of Pharmacy, Medical University of Gdańsk, Dębinki 1, 80-211 Gdańsk, Poland; 2 Department of Environmental Analysis, Faculty of Chemistry, University of Gdańsk, Wita Stwosza 63, 80-308 Gdańsk, Poland

**Keywords:** branched-chain fatty acids, hepatocytes, inflammation, nutrition, obesity, triglycerides

## Abstract

Fatty acids (FAs) are known to play an important role in human metabolism; however, still little is known about the functions of certain FA classes present in blood at relatively low concentrations. Examples of such compounds include branched-chain fatty acids (BCFAs). Recently, lowered BCFAs blood concentration was noticed in obese patients. An inverse correlation was found between serum concentrations of BCFAs and triglyceride levels, as well as C-reactive protein (CRP) concentration. Obesity is the most frequently observed component of metabolic syndrome and both disorders are accompanied by the dysregulation of FAs metabolism. However, not all of them are well understood. Our study is the first attempt at presenting the opposite effects of an *iso*-BCFA (14-methylpentadecanoic acid, 14-MPA) and an *anteiso*-BCFA (12-methyltetradecanoic acid, 12-MTA) on selected genes related to fatty acid synthesis and inflammation: *FASN*, *SREBP1*, *CRP*, and *IL-6* in the HepG2 cell line. We observed lowered expression of *FASN*, *SREBP1*, *CRP*, and *IL-6* in cells treated with 14-MPA in comparison with control cells. In contrast, supplementation with 12-MTA caused opposite effects: increased mRNA levels of *FASN*, *CRP*, and *IL-6*. 12-MTA did not influence *SREBP1* expression. The results of our preliminary study may suggest potential benefits of the supplementation of *iso*-BCFAs in obese patients, for inflammation and hypertriglyceridemia prevention.

## Introduction

Branched-chain fatty acids (BCFAs) are a group of fatty acids (FAs) which are present in human blood in relatively low concentrations; however, they may play an important role in human metabolism [1,2]. Structurally, these compounds are FAs with an additional methyl group attached to the *n*-2 carbon atom (*iso* configuration) or *n*-3 carbon atom (*anteiso* configuration). Some BCFAs, for example, phytanic acid, contain multiple methyl groups. BCFAs are known to enhance cell lipid membrane fluidity [3] and they are also less vulnerable to oxidation in comparison with unsaturated FAs [4]. They can be found in many organisms such as bacteria [5], *Caenorhabditis elegans* [6], mice [7] and rats [8]. BCFAs were also detected in human adipose tissue [9,10]. In adipocytes, they might be synthesized *de novo* from branched-chain amino acids [11]. Nevertheless, the main source of these FAs for humans are dairy products, as ruminant milk is known to be particularly rich in BCFAs [12]. BCFAs with multiple methyl groups may contribute to the pathogenesis of certain diseases, for instance, disorders caused by peroxisome biogenesis defects, connected with the accumulation of BCFAs and very long-chain FAs [13]. Examples of such conditions are Zellweger syndrome [14] and Refsum disease [15]. On the other hand, some monomethyl BCFAs are known to exert anti-cancer properties caused by, for example, the inhibition of fatty acid synthesis and oxidation [16] or the triggering of apoptotic cell death [17]. Some of them exert anti-inflammatory properties, which were observed in neonatal rat models [18]. We have described the already known effects of BCFAs in our recent review paper [2].

Recently, alterations in BCFAs levels were discovered in patients suffering from obesity [1]. Obesity is a disease where an individual’s body mass index (BMI) exceeds 30 kg/m^2^ [19] and, together with hypertension and diabetes, is one of the symptoms indicating metabolic syndrome. Obesity may be connected with changes in blood lipid profile, for example, elevated levels of total cholesterol, triglycerides (TG), and low density lipoprotein cholesterol, as well as decreased levels of high density lipoprotein cholesterol [20] and alterations in serum FA composition [1]. Obesity is also associated with chronic inflammation, indicated by elevated C-reactive protein (CRP) levels [21,22]. The treatment of metabolic syndrome and obesity depends on the severity of the patient’s condition and includes dietary modifications, physical activity, pharmacotherapy, and surgical interventions, i.e., bariatric surgery [23].

Our previous study showed that obese people had lower serum concentrations of *iso*-BCFAs [1]. Moreover, Su et al. found lower total BCFAs in the adipose tissue of obese subjects [9]. After conducting bariatric surgery, the levels of BCFAs returned to values observed in healthy individuals, simultaneously with weight loss [10]. Furthermore, an inverse correlation was found between the concentrations of *iso*-BCFAs and serum concentrations of CRP, insulin and TG [1]. CRP is a widely used diagnostic marker of inflammation [24]. Elevated levels of insulin indicate a risk of insulin resistance and, consequently, diabetes [25]. TG may also serve as a marker of metabolic disorders [26]. The liver is a major source of the synthesis of both TG and CRP in the human body.

However, the above-discussed clinical studies cannot provide evidence of the influence of BCFAs on TG synthesis or the inflammation process. It was previously described that BCFAs may affect several gene expressions, including peroxisome proliferator-activated receptor *α* (*PPARα*) in FaO rat *hepatoma* cells [27] and interleukin-8 (*IL-8*) in human intestinal epithelial Caco-2 cells [28]. Recently, alterations in the expression of genes connected with lipid metabolism and inflammation were observed in white preadipocytes isolated from the adult visceral adipose tissue [29]. Therefore, the aim of our study was to examine the influence of selected *iso*- and *anteiso*-BCFAs on the expression of genes related to FA synthesis and inflammation in the hepatocyte cell line HepG2. We selected the representatives of *iso*- and *anteiso*-BCFA, respectively, 14-methylpentadecanoic acid (14-MPA) and 12-methyltetradecanoic acid (12-MTA), that are present in human blood and have chains of a similar length [1].

## Hypothesis

Taking into consideration that obese patients have lower concentrations of *iso*-BCFAs than healthy individuals and that an inverse correlation was observed between the concentrations of *iso*-BCFAs and serum concentrations of CRP, insulin and TG, we hypothesize that BCFAs may affect the expression of genes related to FAs synthesis and inflammation. Our goal was to assess whether BCFAs may exert potential beneficial effects connected with these processes.

## Materials and methods

### Cell culture

Experiments were conducted using the human hepatocellular carcinoma cell line HepG2, supplied by the American Type Culture Collection (cat. no. HB-8065, ATCC, Manassas, Virginia, U.S.A.). The cell line was selected for its properties connected with lipid metabolism, similar to normal hepatocytes. Cells were maintained in a Minimum Essential Medium (MEM, cat. no. M2279, Sigma Aldrich, St. Louis, Missouri, U.S.A.) supplemented with 10% fetal bovine serum (FBS, cat. no. F9665, Sigma Aldrich), 5 ml of 200 mM L-glutamine (cat. no. G7513, Sigma Aldrich) 5 ml of penicillin/streptomycin solution (100 units/ml penicillin and 100 μg/ml streptomycin, cat. no., P0781, Sigma Aldrich) and 5 ml of 100× non-essential amino acid solution (cat. no. M7145, Sigma Aldrich). Cells were cultured at 37°C in 5% CO_2_ on 60 mm diameter dishes and passaged onto experimental plates according to the supplier’s protocol.

14-MPA and 12-MTA (cat. no. M6781 and M3664, Sigma Aldrich) were dissolved in dimethyl sulfoxide (DMSO, cat. no. D8418, Sigma Aldrich) to a concentration of 50 mM each and mixed with the MEM in order to obtain the following concentrations in the experimental medium: 1, 2, 5, and 10 μM. Twenty-four hours after passaging, when the cells had reached approximately 60–70% confluence, the growth medium was replaced by the experimental medium. Control cells were cultured in parallel in a basic growth medium. After 48 h of incubation with specific concentrations of the selected BCFAs, the cells were collected for RNA isolation and gas chromatography-mass spectrometry (GC-MS) analysis. To examine the effects of 14-MPA, 12-MTA, or DMSO on cell viability, the cells were stained with 0.4% trypan blue (cat. no. T8154, Sigma Aldrich) and counted using the Countess II Automated Cell Counter (Thermo Fisher Scientific, Waltham, Massachusetts, U.S.A.). Observations of cell morphology were conducted using the EVOS XL Core microscope (Thermo Fisher Scientific). Additionally, for higher concentrations of the investigated BCFAs (up to 200 μM) MTT assay was conducted in order to assess metabolic activity of the cells.

### RNA isolation and determination of the mRNA level

Total RNA was extracted using the Direct-zol RNA Miniprep Plus (cat. no. R2070, Zymo Research, Irvine, California, U.S.A.) according to the instruction given by the manufacturer. A reverse transcription of 1 μg of the obtained RNA was performed with the RevertAid First Strand cDNA Synthesis Kit (cat. no. K1621, Thermo Fisher Scientific), in line with the supplier's manual. The mRNA level was measured with the CFX Connect Real-Time system (Bio-Rad, Hercules, California, U.S.A.). The real-time PCR reaction was performed using SYBR Green dye (cat. no. BIO-98005, Bioline, Memphis, Tennessee, U.S.A.). β-Actin and cyclophilin A were chosen as reference genes. The sequences of used primers are presented in Table 1. The relative gene expression was calculated using the ΔΔCt method.

**Table 1:** Sequences of primers used for quantitative PCR

Gene (encoded protein)	Forward primer (5′→3′)	Reverse primer (5′→3′)
*FASN* (fatty acid synthase)	CGCTCGGCATGGCTATCT	CTCGTTGAAGAACGCATCCA
*SREBP1* (sterol regulatory element binding protein 1)	CGGAACCATCTTGGCAACA	GCCGGTTGATAGGCAGCTT
*CRP* (C-reactive protein)	ACTTCCTATGTATCCCTCAAAG	CTCATTGTCTTGTCTCTTGGT
*IL-6* (interleukin 6)	GGTACATCCTCGACGGCATCT	GTGCCTCTTTGCTGCTTTCAC
*ACTB* (β-actin)	AGCACAGAGCCTCGCCTT	CATCATCCATGGTGAGCTGG
*PPIA* (cyclophilin A)	CGTCTCCTTTGAGCTGT	TCGAGTTGTCCACAGTCA

### GC/MS analysis

The total lipids from cell pellets and media were extracted as described by Folch et al. [30]. The extracted fatty acids were derivatized to methyl esters with a 10% boron trifluoride/methanol solution. The fatty acid profiles were analyzed using a GC-EI-MS QP-2010 SE (Shimadzu, Kyoto, Japan), as described previously [31]. All necessary chemicals and reagents were obtained from Sigma Aldrich. Based on the internal standards and the known mass spectra of FAs, the individual BCFAs were identified and the concentrations of specific BCFAs were calculated.

### Statistical analysis

All experiments were performed with at least three repetitions. The statistical analysis was conducted with Statistica v13.3 (StatSoft, Kraków, Poland), using single-factor ANOVA with Tukey’s post-hoc test for unequal group size. Differences between control cells and cells treated with selected fatty acids were considered significant for *P*-values<0.05. Figures were prepared in the Sigma-Plot 11 software (Systat Software Inc., San Jose, California, U.S.A.) and in Microsoft Excel (Microsoft Corporation, Redmond, Washington, U.S.A.).

## Results

### Morphology of cells

**Figure 1:**
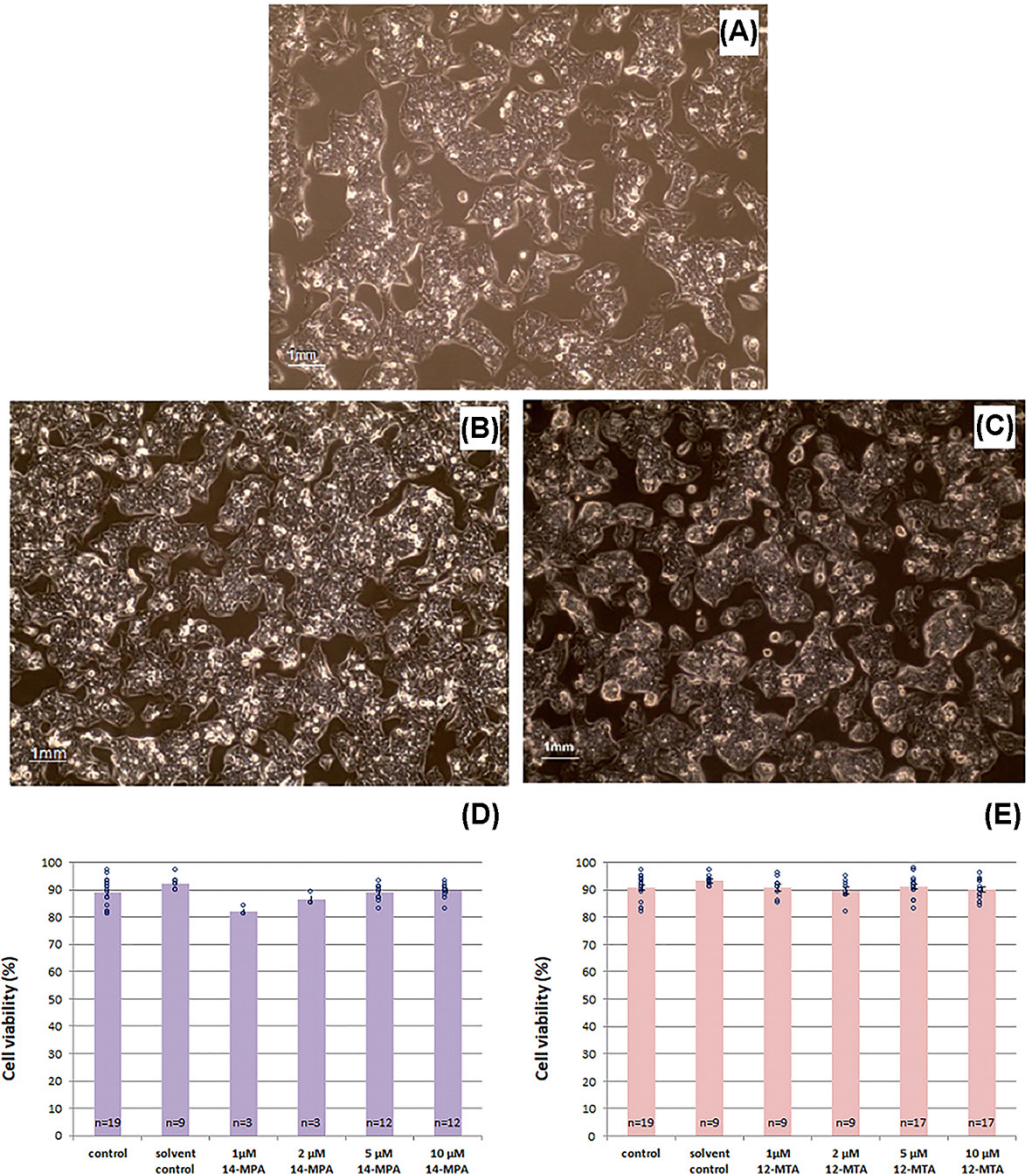
HepG2 cells morphology (magnification: 400×) and viability (**A**) HepG2 cells non-treated with BCFA. (**B**) HepG2 cells after treatment with 10 µM 14-MPA. (**C**) HepG2 cells after treatment with 10 µM 12-MTA. (**D**) Cell viability after treatment with 14-MPA. (**E**) Cell viability after treatment with 12-MTA. ○ - individual data points. Three independent experiments were performed and a total number of repetitions (*n*), summed from all experiments, is indicated in the bars.

The treatment with 14-MPA, 12-MTA, and DMSO did not affect cell viability. HepG2 cells grew in a monolayer, in clusters of adjacent cells. No changes in cell morphology (Figure 1A–C) and in their viability (Figure 1D,E) were observed during the time of the experiment. Additionally, MTT assay revealed no changes in the metabolic activity of the cells treated with higher concentrations of the investigated BCFAs.

### Uptake of BCFAs by HepG2 cells from the experimental media

**Figure 2:**
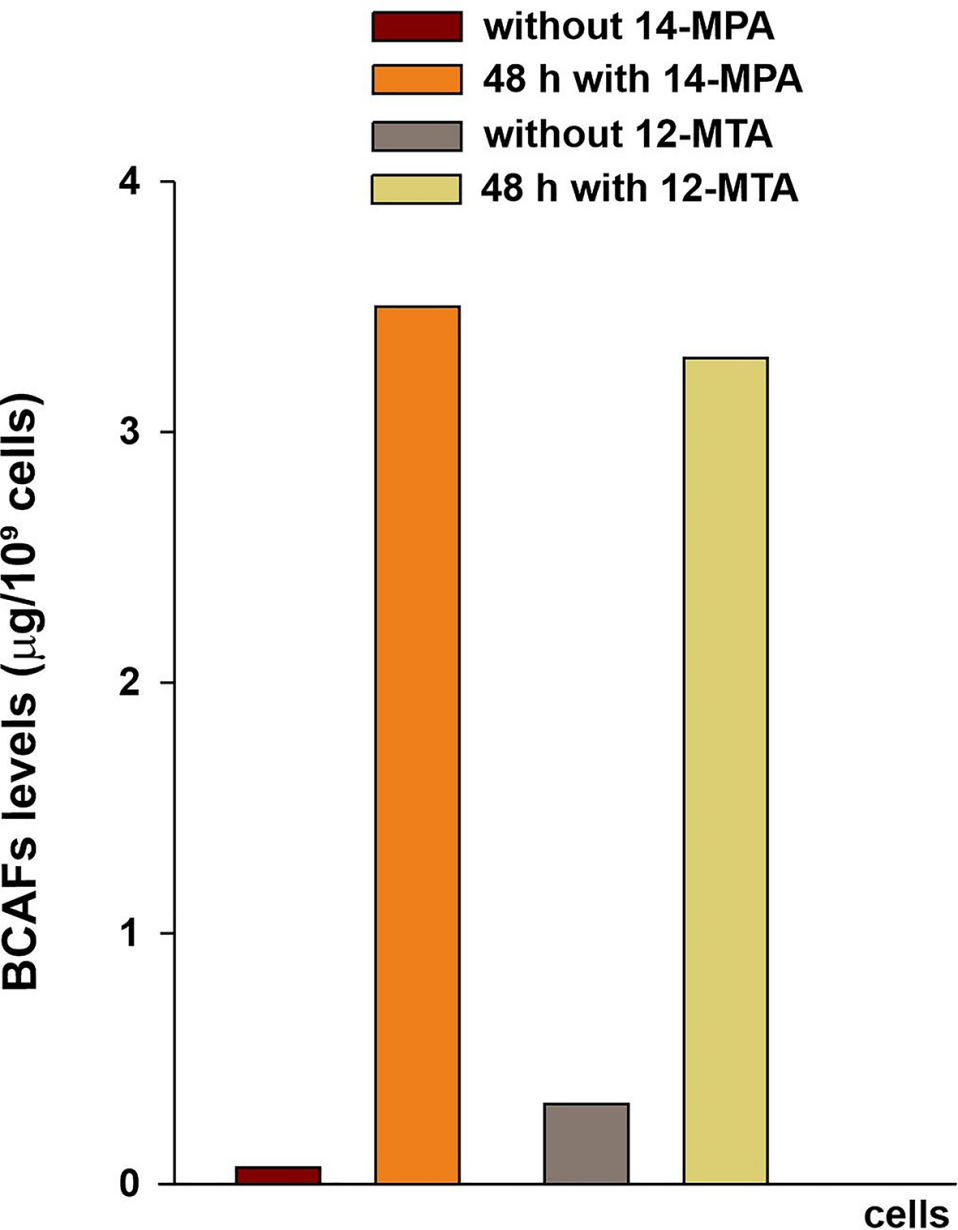
Uptake of BCFAs by HepG2 cells from the experimental media Concentration of BCFAs in HepG2 cells collected after 48 h of incubation with 10 µM 14-MPA or 10 µM 12-MTA. The analysis conducted in the samples prepared by pooling three individual cell cultures from a single experiment.

To estimate the uptake of BCFAs by HepG2 cells, the content of these fatty acids was measured in the cells before and after the incubation with 10 µM 14-MPA and 10 µM 12-MTA. After 48 h of incubation high levels of these BCFAs were observed in the treated cells in comparison with the control cells (Figure 2): approximately 50 times higher in the cells treated with 14-MPA and approximately 10 times higher in the cells treated with 12-MTA. At the same time, the contents of both supplemented BCFAs were similar in the treated cells. These results suggest that HepG2 cells effectively uptake BCFAs from the medium.

Moreover, we have observed a significant increase in BCFAs longer by two atoms of carbon – 16-methylheptadecanoic acid (*iso*-BCFA) in the cells treated by 10 µM 14-MPA (0.28 µg/10^9^ cells vs. 0.002 µg/10^9^ cells in the control culture), and 14-methylhexadecanoic acid (*anteiso*-BCFA) in the cells treated by 12-MTA (2.4 µg/10^9^ cells vs. 0.16 µg/10^9^ cells in the control culture). BCFAs with chains longer than 18 carbons were present in the treated cells in trace amounts.

### Influence of 14-MPA and 12-MTA on the expression of genes related to fatty acid synthesis in HepG2 cells

**Figure 3:**
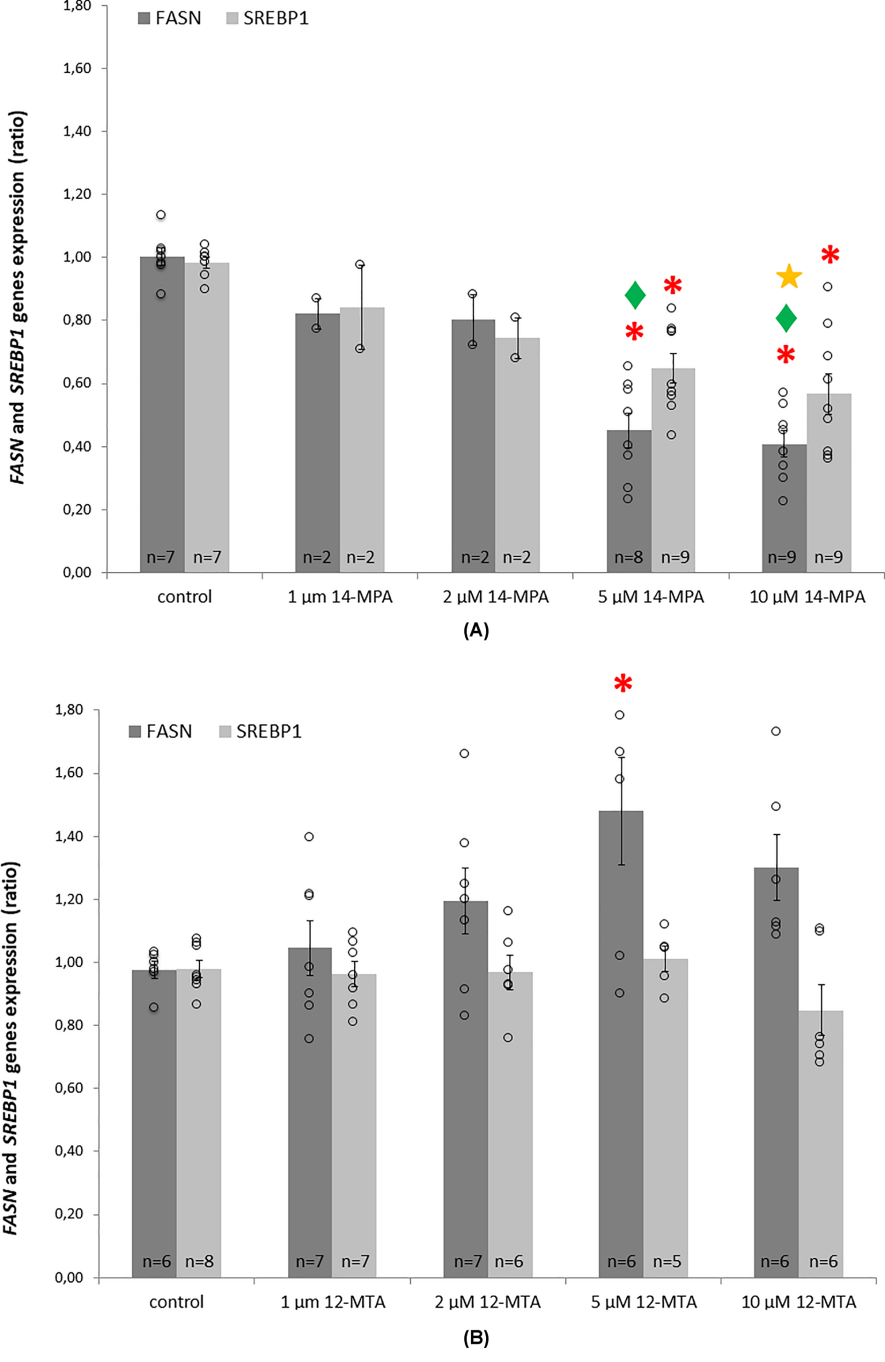
The mRNA levels of FASN and SREBP1 in HepG2 cells treated with 14-MPA and 12-MTA (**A**) Cells treated with 14-MPA. (**B**) Cells treated with 12-MTA. Data in (**A,B**) are presented as means ± SEM; statistical test used: single-factor ANOVA with Tukey post-hoc test for unequal group size; *P-value<0.05 in comparison with control cells, ♦*P*-value<0.05 in comparison with cells treated with 1 μM BCFA; *P-value<0.05 in comparison with cells treated with 2 μM BCFA; ○ - individual data points. Three independent experiments were performed and a total number of repetitions (*n*), summed from all experiments, is indicated in the bars. Concentrations 1 and 2 μM of 14-MPA were used in two of the independent experiments only, with one repetition per experiment.

After incubation with 14-MPA, significantly lower mRNA levels of *FASN* (a gene encoding fatty acid synthase – FASN) were noted after treatment with 5 μM and 10 μM concentrations of this BCFA (Figure 3A), which are similar to the physiological levels of this fatty acid in the serum of healthy, non-obese patients. Lower concentrations (1μM, 2 μM) of 14-MPA exerted a weaker effect. A similar relationship was observed for *SREBP1* (a gene encoding sterol regulatory element binding protein 1 – SREBP1): the lowest mRNA levels were observed in cells treated with 5 μM and 10 μM 14-MTA. No effect was noticed after the administration of lower concentrations of this BCFA (Figure 3A).

By contrast, treatment with 12-MTA caused a concentration-dependent increase in mRNA levels of *FASN* in hepatocytes. Significantly higher expression was observed in cells treated with 5 μM of 12-MTA, but those treated with 10 μM of 12-MTA showed a similar trend (*P*=0.269) (Figure 3B). The mRNA levels of *SREBP1* after 12-MTA treatment were not significantly changed in comparison with the control cells (Figure 3B).

### Influence of 14-MPA and 12-MTA on the expression of genes related to inflammation

**Figure 4:**
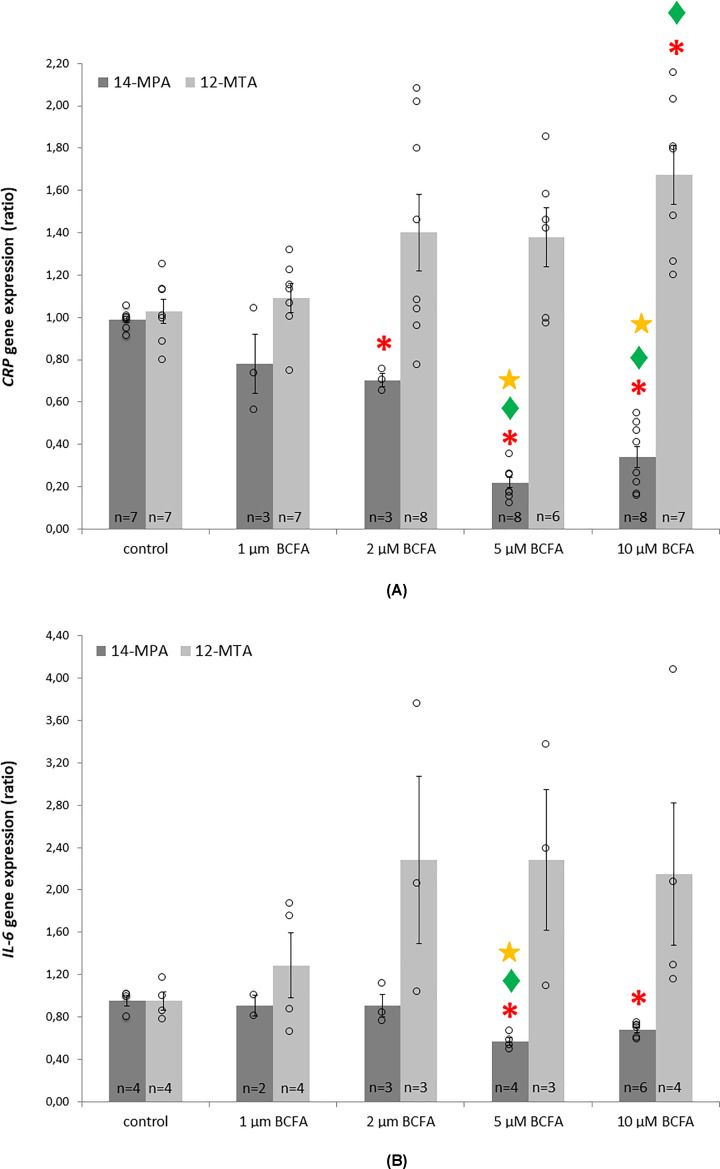
The mRNA levels of genes related to inflammation in HepG2 cells treated with 14-MPA and 12-MTA (**A**) CRP mRNA levels. (**B**) IL-6 mRNA levels. Data in (**A,B**) are presented as means ± SEM; statistical test used: single-factor ANOVA with Tukey post-hoc test for unequal group size; *P-value<0.05 in comparison with control cells, ♦*P*-value<0.05 in comparison with cells treated with 1 μM BCFA; *P-value<0.05 in comparison with cells treated with 2 μM BCFA. Three independent experiments were performed and a total number of repetitions (*n*), summed from all experiments, is indicated in the bars. For *IL-6* gene expression analysis, one repetition for 1 μM of 14-MTA was excluded (outlier), so the total number of repetitions in this case is two.

The administration of 14-MPA resulted in a decrease in *CRP* gene expression, compared with the control cells. The strongest effect was observed for 5 and 10 μM. In turn, treatment with 12-MTA resulted in a concentration-dependent increase in *CRP* expression in comparison with the control cells (Figure 4A). The anti-inflammatory effects of 14-MPA were also supported by decreased mRNA levels of *IL-6* after the treatment of HepG2 hepatocytes by this BCFA. Similarly, the strongest effect was noted for 5 and 10 μM. By contrast, *IL-6* mRNA level tended to increase after 12-MTA treatment, which further confirms the opposing effects of *iso*- and *anteiso*-BCFAs (Figure 4B).

## Discussion

Human liver plays a crucial role in lipid homeostasis. Hepatocytes are engaged in the uptake, synthesis and esterification of FAs as well as the release of TG into the blood [32]. The liver is also a major organ producing and releasing CRP. Our study was focused on the impact of BCFAs on FAs metabolism and the inflammatory response in hepatocytes. BCFAs, although present in human blood in low amounts, are known to exert beneficial health effects, which include maintaining lipid homeostasis [33] and anti-inflammatory properties [18]. However, the exact mechanisms of their actions remain unclear. Therefore, our findings are the initial evidence to elucidate the molecular mechanism of the influence of BCFAs on TG synthesis and inflammation in the liver.

The normalization of lipid metabolism as well as blood lipid composition is essential for obese patients, suffering from metabolic syndrome. Inverse correlations between serum concentrations of *iso*-BCFAs and serum TG concentrations in obese individuals [1] may suggest the possible impact of these FAs on maintaining lipid homeostasis. As mentioned above, BCFAs have the potential to influence gene expression, for example, *PPARα* in rat hepatoma cells [27] and *IL-8* in human intestinal epithelial cells [28]. They are also capable of binding the nuclear receptors involved in modulating the transcription of genes [34].

For our research, we selected two genes encoding proteins engaged in FA metabolism (*FASN* and *SREBP1*). FASN is an enzymatic complex responsible for the synthesis of FAs, which serves as a substrate for TG production; hence, there is a strong association between FASN and TG levels. Lowered *FASN* expression may contribute to a decrease in the synthesis and release of TG from hepatocytes. *SREBP1* serves as an upstream regulator of FASN [35].

In accordance with our research, treatment with *iso*-BCFAs in a selected range of concentrations caused a dose-dependent decrease in the expression of selected genes related to fatty acid synthesis in hepatocytes. It can be speculated that *iso*-BCFAs attenuate FASN synthesis, which might therefore result in lower TG levels. Thus, lowered *iso*-BCFAs in obese subjects may lead to increased FAs and, consequently, TG synthesis in their livers. By contrast, we obtained the opposite results for *anteiso*-BCFAs; in comparison with *iso*-BCFAs, *anteiso*-BCFAs appeared to increase the mRNA level of *FASN*.

Additionally, we demonstrated that *iso*-BCFAs influence the expression of *SREBP1*, a transcriptional factor involved in, amongst others, cholesterol and fatty acids synthesis. It plays a key role in the transcriptional regulation of the *FASN* gene [36]. SREBP1 binds to the *FASN* gene promoter, enhancing its expression [37]. Administering 14-MPA to HepG2 cells caused a decrease in the *SREBP1* mRNA level, and this is in line with a decrease in the *FASN* mRNA level; therefore we conclude that lowered *FASN* expression is likely caused by a decrease in *SREBP1* expression. *Anteiso*-BCFAs did not influence *SREBP1* expression; nevertheless, the mRNA level of *FASN* in cells treated with *anteiso*-BCFAs turned out to be significantly elevated in comparison with the control cells. This suggests the contribution of different signaling pathways in *FASN* regulation in response to *anteiso*-BCFAs.

To the best of our knowledge, we are the first to determine that *iso*-BCFAs can attenuate the expression of genes encoding proteins involved in lipid synthesis in hepatocytes. An elevated TG concentration may lead to atherosclerosis development, consequently increasing the risk of stroke and myocardial infarction. It is also one of the characteristics of obesity. Therefore, we suggest that the normalization of *iso*-BCFA concentration may contribute to an improvement in the lipid profile in obese individuals. This is supported by decreased levels of TG and an increase in the concentration of *iso*-BCFAs in obese individuals after bariatric surgery and following weight loss. Although the levels of *anteiso*-BCFAs were also increased after bariatric surgery, which may abolish the effect of increased *iso*-BCFAs, in contrast with *iso*-BCFAs, *anteiso*-BCFAs did not reach the levels observed in lean subjects [10].

Obesity is also associated with high levels of CRP. In our study, we determined that the investigated *iso*-BCFA has the potential to lower *CRP* gene expression, which might be a possible beneficial effect for obese individuals, as BMI values are strongly correlated with serum CRP concentration [22]. Similarly, *iso*-BCFA decreased the expression of *IL-6* gene in HepG2 cells. The *anteiso*-BCFA used in our experiment turned out to enhance *CRP* and *IL-6* genes expression. Again, these effects may explain the simultaneous increase in *iso*-BCFAs and decrease in CRP in obese subjects after bariatric surgery [10].

Considering that after treating the cells with 14-MPA and 12-MTA we observed a significant increase in BCFAs longer by two atoms of carbon together with the fact that in mammalian cells BCFAs may be further metabolized, e.g., elongated to longer BCFAs [38], we also presume that the cells could have been exposed to the effects of not only 14-MPA or 12-MTA but also *iso*-BCFAs or *anteiso*-BCFAs longer by two carbon atoms. However, BCFAs with chains longer than 18 carbons were present in the treated cells in trace amounts. The fate of BCFAs incorporated into the cells needs to be further investigated.

The determination of the exact molecular mechanism of the anti-inflammatory effect of *iso*-BCFAs was beyond the scope of our study; however, in connection with the current literature, we could speculate on the potential role of the mitogen-activated protein kinase/nuclear factor kappa-B (MAPK/NFκB) signaling pathway. The involvement of MAPK/NFκB in the FA mechanism of action in suppressing inflammation was already described for various FAs, for example, eicosapentaenoic acid [39]. Another *iso*-BCFA, 13-methyltetradecanoic acid, which has a similar chain length to 14-MPA, has the potential to regulate MAPK phosphorylation as well [40]. Therefore, we speculate that the anti-inflammatory effect of the investigated *iso*-BCFA might be connected with the MAPK/NFκB pathway, and this issue needs further investigation in order to determine the detailed molecular mechanism.

The limitation of our study is the fact that the expression of the selected genes was assessed only on mRNA level, not protein. Also, the experiments were conducted *in vitro*, therefore the results should be treated as preliminary and confirmed in an animal model, before planning any future studies in human. Moreover, we have tested the effect of only two BCFAs and the number of repetitions was relatively low.

In conclusion, our study showed the opposite effects of representatives of *iso*-BCFAs and *anteiso*-BCFAs on the expression of genes coding FASN and CRP. Further research is needed to verify the effects of other *iso*- and *anteiso*-BCFAs present in human blood. Moreover, the results of our study may suggest the potential of the supplementation of *iso*-BCFAs in obese patients to prevent inflammation and hypertriglyceridemia. To the best of our knowledge, this is the first study which shows the effect of long-chain BCFAs on hepatocytes.

## Testable hypotheses and direction for future research

Our previous studies showed that obese patients have lower concentrations of *iso*-BCFAs than healthy individuals. Furthermore, an inverse correlation was observed between the concentrations of *iso*-BCFAs and serum concentrations of CRP (inflammation marker), insulin and TG (connected with metabolic disorders) [1]. Taking into consideration that the liver is the main organ responsible for the synthesis of TG and CRP for humans, and also that BCFAs might influence the genes expression [28], we hypothesized that BCFAs may affect the expression of genes related to FAs synthesis and inflammation. In the study described in this paper, our hypothesis has been confirmed on the mRNA level.

The impact of BCFAs on human metabolism and inflammation process is still not a widely explored topic. Further research is needed to confirm the results obtained in our study. Experiments on animal models using food supplemented with BCFAs may lead to better understanding of the role of BCFAs in the mentioned processes, and the outcomes of the administration of *iso*-BCFAs to animals might serve as a basis for the design of future studies concerning the potential beneficial effects of supplementation of these fatty acids to the obese patients, or patients with other metabolic disorders.

## Data Availability

Data to be shared upon request (corresponding author: paulina.st@gumed.edu.pl).

## Abbreviations

12-MTA: 12-methyltetradecanoic acid
14-MPA: 14-methylpentadecanoic acid
BCFA: branched-chain fatty acid
CRP: C-reactive protein
FASN: fatty acid synthase
*IL-8*: interleukin-8
MAPK/NFκB: mitogen-activated protein kinase/nuclear factor kappa-B
*PPARα*: peroxisome proliferator-activated receptor *α*
